# One-Pot Synthesis of Hydrophilic and Hydrophobic N-Doped Graphene Quantum Dots via Exfoliating and Disintegrating Graphite Flakes

**DOI:** 10.1038/srep30426

**Published:** 2016-07-25

**Authors:** Na-Jung Kuo, Yu-Syuan Chen, Chien-Wei Wu, Chun-Yuan Huang, Yang-Hsiang Chan, I-Wen Peter Chen

**Affiliations:** 1 Department of Applied Science, National Taitung University, 369, Sec. 2, University Road, Taitung City 95092, Taiwan; 2 Department of Chemistry, National Taiwan University, 1, Sec. 4, Roosevelt Road, Taipei, 10617, Taiwan; 3 Department of Chemistry, National Sun Yat-sen University, 70 Lien Hai Road, Kaohsiung, 80424, Taiwan

## Abstract

Graphene quantum dots (GQDs) have drawn tremendous attention on account of their numerous alluring properties and a wide range of application potentials. Here, we report that hydrophilic and hydrophobic N-doped GQDs can be prepared via exfoliating and disintegrating graphite flakes. Various spectroscopic characterizations including TEM, AFM, FTIR, PL, XPS, and Raman spectroscopy demonstrated that the hydrophilic N-doped GQDs (IN-GQDs) and the hydrophobic N-doped GQDs (ON-GQDs) are mono-layered and multi-layered, respectively. In terms of practical aspects, the supercapacitor of an ON-GQDs/SWCNTs composite paper electrode was fabricated and exhibited an areal capacitance of 114 mF/cm^2^, which is more than 250% higher than the best reported value to date for a GQDs/carbon nanotube hybrid composite. For IN-GQDs applications, bio-memristor devices of IN-GQDs-albumen combination exhibited on/off current ratios in excess of 10^4^ accompanied by stable switching endurance of over 250 cycles. The resistance stability of the high resistance state and the low resistance state could be maintained for over 10^4^ s. Moreover, the IN-GQDs exhibited a superior quantum yield (34%), excellent stability of cellular imaging, and no cytotoxicity. Hence, the solution-based method for synchronized production of IN-GQDs and ON-GQDs is a facile and processable route that will bring GQDs-based electronics and composites closer to actualization.

Although graphene is a zero-band gap material, its surface-functionalized domains have proved to contain a certain band gap depending upon their shape, size, and edge states, because of either quantum confinement^1^ or edge effect^2^,^3^. Recently, graphene quantum dots (GQDs) have drawn significant attention on account of their numerous alluring properties and a wide range of application potentials^4^,^5^,^6^,^7^,^8^,^9^. One of the most noteworthy topics of the GQDs is their potential for being used in place of frequently used inorganic hybridized materials including toxic/expensive heavy metals, where their versatile tunability properties along with solution-processing technology can be used in various applications^8^,^10^. Furthermore, the band-gap of the GQDs can be artificially engineered by tuning conjugation length and surface functionalities^11^,^12^,^13^,^14^, leading to a number of novel properties such as photoluminescence (PL) of higher intensity and anti-photobleaching ability. GQDs are nanoscale structures of mono- or multi-layered graphene consisting of a uniform hexagonal lattice of *sp*^2^ carbon hybridization incorporated with heteroatomic functional groups^7^,^15^. According to previous studies, most synthesized GQDs exhibit hydrophilic properties, and therefore, are limited to water-based applications^2^,^5^,^16^,^17^,^18^,^19^,^20^,^21^,^22^,^23^.

Considering that wet-coating technology, commonly adopted in the assembly of state-of-the-art advanced electronic and energy storage devices, has relied on organic media, such “hydrophilicity” definitely limits the previously-mentioned GQDs applications in the field of organic-based electronic and energy storage devices because of their incompatible solubility in common organic solvents. In this regard, it has been difficult to make hydrophilic GQDs compatible with other components of organic-based applications, presumably owing to severe aggregation between hydrophilic GQDs while being turned into film or solid forms. Some alternative methods for instant acid-free exfoliation of graphite nanoparticles^24^, conjugation of polyphenylene dendrimers^13^, cage-opening of C_60_ 25, and hexa-peri-hexabenzocoronene polymerization^21^ have been established; however, their applications are limited owing to complicated reaction procedures, low product yield, the requirement for special vacuum equipment, and so on. Recently, Kwon *et al*. demonstrated that hydrophobic GQDs with aliphatic side chains, giving superior organic solubility and no desirable agglomeration, were synthesized via amidative cutting of tattered graphite for electroluminescence application^26^. However, the chemicals and solvents used are highly toxic and require special care during chemical reactions. Therefore, it is important to develop an environmentally-friendly, simple route by which hydrophobic GQDs may be synthesized. According to the aforementioned information, both hydrophobic GQDs and hydrophilic GQDs possess their unique properties and superior performance qualities in the fields of sensing^8^, bio-labeling^7^,^10^ and optoelectronics^20^,^26^, and there are many related applications in primary trials such as energy storage applications.

With these points in mind, it has become necessary to find out what is needed. The aqueous-based synthesis needs to result in the preparation of GQDs with hydrophilic and hydrophobic properties. Moreover, the solvents used should be environmentally-friendly and should not need further chemical post-treatment. In addition, it should be easy to handle, and compatible with common, safe, green solvents. In this paper, we demonstrate a simple, green route for the direct synthesis of hydrophilic N-doped GQDs (IN-GQDs) and hydrophobic N-doped GQDs (ON-GQDs) via the one-pot synthesis method. The potential utility of the as-prepared N-doped GQDs was demonstrated for supercapacitor, inkjet printing, bio-memristor and bio-imaging applications.

## Results and Discussion

**Figure 1:**
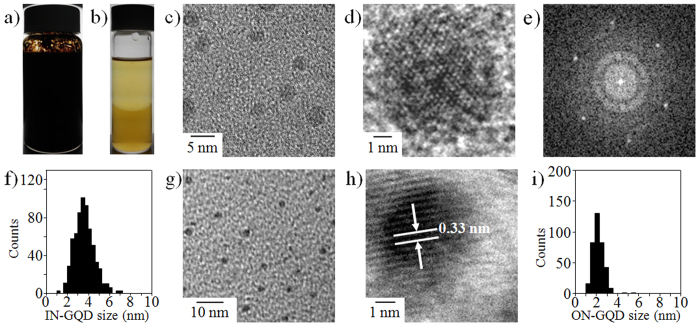
Synthesis and characterization of N-doped GQDs. (**a**) Photograph of the as-synthesized N-doped GQDs solution. (**b**) Dilution of the as-synthesized N-doped GQDs solution. Upper layer: ON-GQDs; bottom layer: IN-GQDs. TEM image of (**c**) IN-GQDs and (**g**) ON-GQDs. HR-TEM image of (**d**) IN-GQDs and (**h**) ON-GQDs. (**e**) 2D FFT image of the IN-GQDs. Histograms of lateral size distributions of (**f**) IN-GQDs and (**i**) ON-GQDs.

According to our previous studies, graphite can be exfoliated into graphene with the assistance of pyridinium tribromine^27^,^28^, resulting in the pyridinium-assisted exfoliated mono-layer graphene sheets (hydrophilic behavior) solution with a size of several hundred nanometers and undissolvable graphite flakes (hydrophobic behavior) floating on top of the pyridinium-assisted exfoliated single-layer graphene sheets solution (Figure S1). The exfoliated graphene sheets (with the help of the adsorbed pyridinum) became soluble in water and exhibited hydrophilic behavior. The undissolvable graphite flake behavior (hydrophobic behavior) could be attributed to the formation of concentrated suspension leaving the surplus solute floating on the top. Then, a doping source of nitrogen atoms, polyethylenimine (PEI), was subsequently added to the aqueous solutions, and the mixtures were involved in hydrothermal reactions at 230 °C and 500 psi for 8 h in a stainless autoclave reactor. The hydrothermal synthesis method has been widely utilized in doping graphene-related materials to prepare N-doped GQDs^29^,^30^. After hydrothermal reaction, the IN-GQDs (bottom layer) and ON-GQDs (upper layer) could be successfully prepared, as shown in Fig. 1a. At centrifugation speeds of 2000 to 12,000 rpm, the IN-GQDs solution exhibited the same color (Figure S2), which means that the IN-GQDs were well-dispersed and their sizes were in the range of nanometers. Moreover, the IN-GQDs and ON-GQDs solutions remained homogeneous even after standing for more than a year on the experimental bench, and they were without recognizable precipitation. To clearly understand the dissolution of IN-GQDs and ON-GQDs in different solvents, we placed 50 μL of each from the as-prepared high concentration N-doped GQDs solution (Fig. 1a) in a glass vial. Then, 2 mL of water and 2 mL of toluene were added. Fig. 1b shows the diluted IN-GQDs (bottom layer) and ON-GQDs (upper layer) with a pale yellow color in the water and toluene.

Transmission electron microscope (TEM) images show that the IN-GQDs (Fig. 1c) and ON-GQDs (Fig. 1g) had a uniform size of about 3.6 ± 0.9 (Fig. 1f) and 2.1 ± 0.6 nm (Fig. 1i), respectively. The 2D fast Fourier transform (FFT) pattern of representative IN-GQDs is shown in Fig. 1e, and the corresponding high-resolution TEM (HR-TEM) image is shown in Fig. 1d. The lattice fringes of the IN-GQDs (Fig. 1d) with a d spacing of 0.21 nm fit with the (100) facet of the graphene^31^,^32^,^33^. The lattice fringes of the ON-GQDs (Fig. 1h) with a d spacing of 0.33 nm fit well with the graphite interlayer spacing^34^,^35^. In order to measure the thickness of the IN-GQDs and ON-GQDs, tapping-mode atomic force microscopy (TM-AFM) characterization was performed. The measured thickness of the IN-GQDs and ON-GQDs was centered around 1 nm and 3 nm, respectively, as shown in Figure S3a,b. According to the analyses of the TEM images and statistical results for thickness, the IN-GQDs and ON-GQDs exhibited nearly mono-layer and multi-layered graphene dots, respectively.

**Figure 2:**
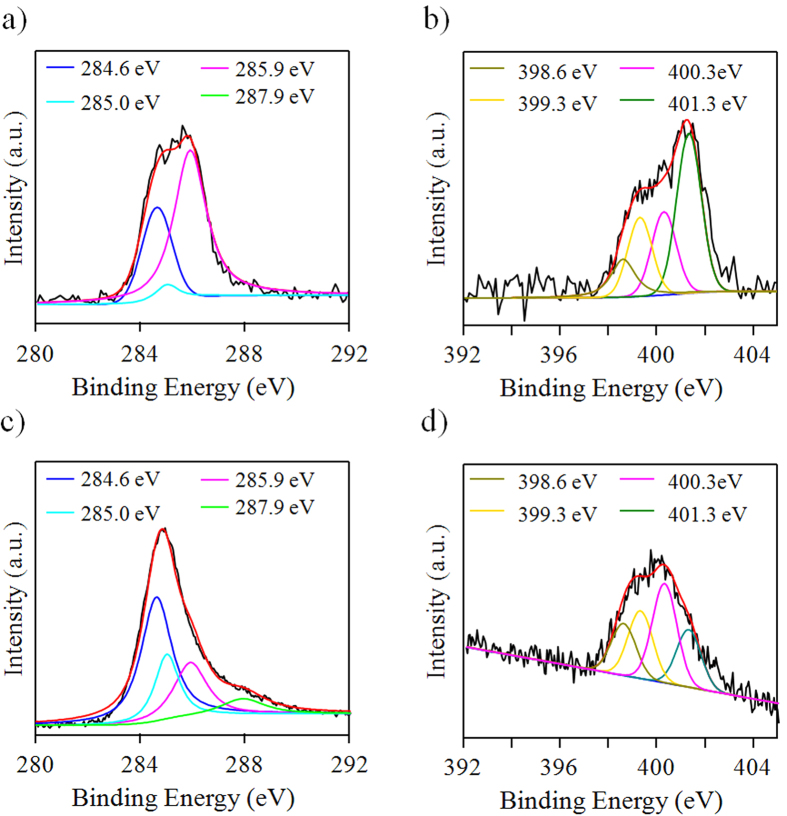
High resolution XPS characterization of N-doped GQDs. (**a**) C 1s and (**b**) N 1s spectrum of IN-GQDs. (**c**) C 1s and (**d**) N 1s of ON-GQDs.

**Figure 3:**
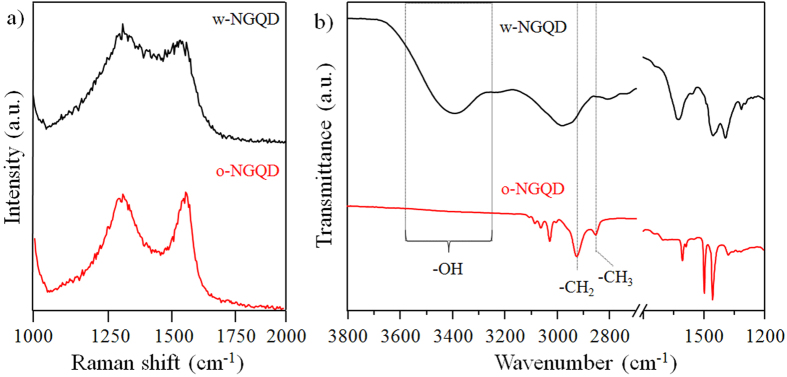
(**a**) Raman, (**b**) FTIR spectra of the indicated N-doped GQDs.

X-ray photoelectron spectroscopy (XPS) was consequently carried out to determine the chemical composition of the as-prepared N-doped GQDs. The high resolution C 1s spectrum of the IN-GQDs (Fig. 2a) can be deconvoluted into several peaks that correspond to C=C (284.6 eV), defective C=C (285.0 eV), C=N (285.9 eV), and C–N (287.9 eV) functional groups. The I_284.6_/I_285.0_ ratio of the IN-GQDs (Fig. 3a) was higher than that of the ON-GQDs (Fig. 2c). This result indicates that the IN-GQDs were of relatively larger fragments than the ON-GQDs^36^. The peak of C 1s 285.9 eV of the IN-GQDs (Fig. 2a) and ON-GQDs (Fig. 2c) indicate that the C–N bondings were generated during N-GQDs synthesis reaction. Figure 2b,d show that the peak being centered at 401.3 eV is attributed to the N 1s of the N−C bond of graphene-like nitrogen, indicating that those decomposed of pyridinium and/or PEI were attached to the aromatic rings of the IN-GQDs. The minor peaks at 398.6, 399.3, and 400.3 eV are ascribed to pyridine derivative, amide N (C–N), and doping N, respectively^37^,^38^,^39^. Therefore, it can be concluded that the as-synthesized N-doped GQDs were composed of *sp*^*2*^ aromatic and aromatic C–N species derived by PEI molecules and pyridinium derivatives along with abundant oxygen- and nitrogen-containing functional groups on the IN-GQDs and ON-GQDs surfaces.

**Figure 4:**
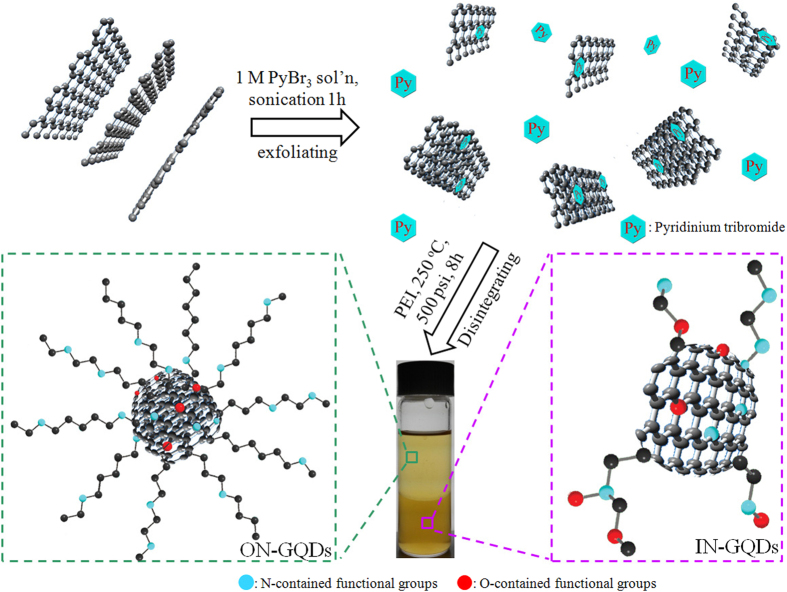
Synthetic scheme for preparation process of IN-GQDs and ON-GQDs. N-contained and O-contained sites are shown as cyan and red dots, respectively. Image is not to scale.

With Raman spectroscopy, we noticed that the G-band and D-band arose from the in-plane of *sp*^*2*^ carbons and *sp*^*3*^-hybridised carbons, respectively. Therefore, the intensity ratio of I_D_/I_G_ is a reliable indicator with which to verify changes from *sp*^*2*^ carbons into defective forms of *sp*^*3*^ configuration in graphene. Figure 3a shows that the I_D_/I_G_ ratios of the IN-GQDs and ON-GQDs are close to 1.0. This value is higher than that of the pristine graphene, probably because IN-GQDs and ON-GQDs have a number of edges and surface functional groups^26^. Attenuated total reflectance fourier transform infrared (ATR-FTIR) spectroscopy is a powerful tool used in observing the surface functionalization of the IN-GQDs and ON-GQDs after disintegrating the exfoliated solution. In Fig. 3b, amide C-N bending (1452 cm^−1^) and O-H bending (~1380 cm^−1^) bonds strongly indicate that oxygen-contained solvents and nitrogen-contained molecules were possibly formed with amine, amide, and hydroxyl groups for the N-doped GQDs^26^,^40^. Although the ATR-FTIR spectrum of the ON-GQDs shown in Fig. 3b looks similar to that of the IN-GQDs at the fingerprint domain, there are significant differences at higher wavenumber regions. A band centered around 3400 cm^−1^ indicates the stretching mode of the hydrogen bonded hydroxyl group^31^. Interestingly, no observable hydrogen bonded hydroxyl group was detected for the ON-GQDs. Moreover, the stretching vibrations of -CH_2_ and -CH_3_ of the ON-GQDs were located around 2922 and 2851 cm^−1^, respectively, demonstrating that the alkyl chains became more densely packed around the surface of the ON-GQDs than the pure PEI molecules (Figure S4) and the IN-GQDs (Fig. 3b)^41^,^42^. Therefore, the hydrophobic property of the ON-GQDs could possibly be attributed to the ordered packing of the alkyl chains and less hydrophilic functional groups on the surfaces. This result is similar to that seen in the chemical functionalization method^26^,^43^,^44^. According to the aforementioned characterization, Fig. 4 shows a proposed scheme for one-pot synthesis of the N-doped GQDs.

### Application of ON-GQDs for a supercapacitor

**Figure 5:**
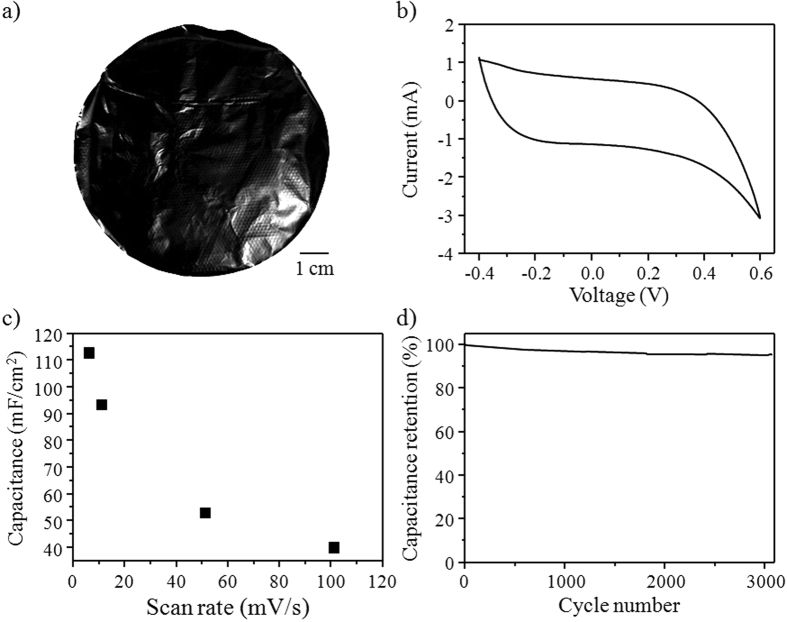
(**a**) Photograph of free-standing composite paper of ON-GQDs/SWCNTs. (**b**) CV curve of ON-GQDs/SWCNTs composite paper with a scan rate of 10 mV/s. (**c**) Areal capacitance versus scan rate for ON-GQDs/SWCNTs composite paper. (**d**) Capacitance retention after 3000 cycles in 1 M KOH.

According to previous studies, N-doped GQDs could be an attractive candidate for optoelectronic devices and catalysts. Unfortunately, other interesting N-doped GQDs applications, such as energy storage devices, have remained rarely explored^45^. Hence, we demonstrate an all carbon-based supercapacitor based on composites of ON-GQDs by a pretreated single-walled carbon nanotubes (SWCNTs) paper as the scaffolding. Therefore, the ON-GQDs could directly adsorb to the side wall of the SWCNTs through hydrophobic-hydrophobic interaction. The flexible ON-GQDs/SWCNTs composite paper (Fig. 5a) was prepared and used as the working electrode in a three-electrode system. Its electrochemical performance was evaluated by cyclic voltammetry (CV) in a 1 M KOH solution with a potential window of −0.4 to 0.6 V (vs. Ag/AgCl). Figure S5 shows that the capacitance of the pristine SWCNTs is ~15 mF/cm^2^. Fig. 5b shows the CV curve for the ON-GQDs/SWCNTs composite paper at a scanning rate of 10 mV/s. The ON-GQDs/SWCNTs composite supercapacitor exhibited typical double-layer capacitive behavior at scan rates ranging from 5 to 100 mV/s (Fig. 5c). As pointed out by Gogotsi *et al*.^46^, areal capacitance is a more reliable performance metric for supercapacitor devices in comparison to gravimetric capacitance. Therefore, the ON-GQDs/SWCNTs composite paper supercapacitors were evaluated in the unit of mF/cm^2^. The areal capacitance of the ON-GQDs/SWCNTs composite paper supercapacitor was 114 mF/cm^2^, representing a more than 700% increase over that of the pristine SWCNTs paper electrode. This result is more than 250% higher than the method of electrodeposited GQDs on carbon nanotube networks^45^. Moreover, the performance of the ON-GQDs/SWCNTs is superior than results from previous studies among carbon-related supercapacitors, for instance, with regards to the chemical vapor deposition graphene sheets (80 μF/cm^2^)^47^, reduced multi-layered graphene oxide (394 μF/cm^2^)^47^, and tri-layered graphene oxide supercapacitor devices (510 μF/cm^2^)^48^. It is thought that the ON-GQDs can efficiently adsorbed onto the SWCNTs surfaces for enhanced charge storage in the ON-GQDs/SWCNTs composite paper electrode. Figure 5d shows that after 3000 consecutive cycles, the specific capacitance of the ON-GQDs/SWCNTs composite paper electrode still retained 95% of its initial capacitance, indicating that the ON-GQDs/SWCNTs composite paper supercapacitors were highly stable under the testing conditions. These results indicate that the edge-abundant ON-GQDs possibly cause enhanced electrode capacitance owing to better accessibility of electrolyte ions via free end edges because the edge sites possess the capacity to accumulate more charges than the SWCNTs surface.

### Application of IN-GQDs for inkjet printing

**Figure 6:**
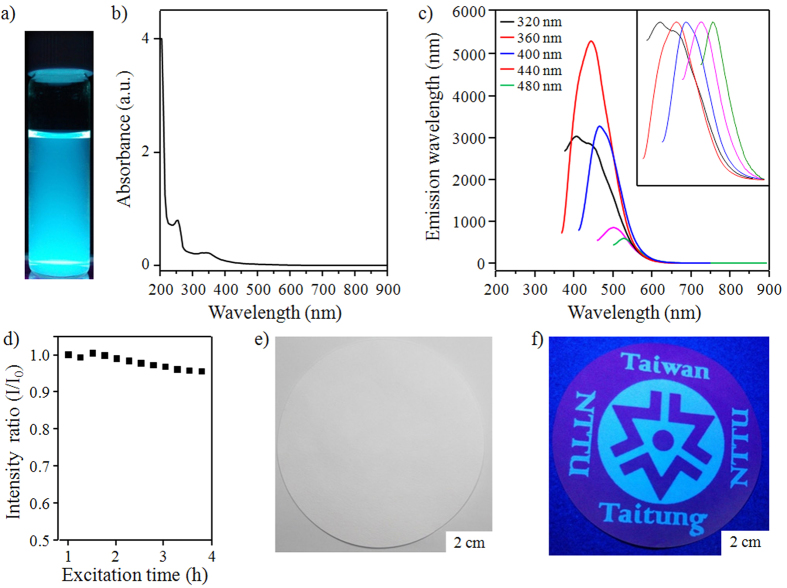
Photophysical properties of IN-GQDs. (**a**) PL of the dilute solution kept at ambient condition and excited by a 365 nm handheld UV lamp. (**b**) UV-vis absorption spectrum. (**c**) PL spectra of the dilute solution at different excitation wavelengths. (**d**) Photobleaching test. (**e**) Optical photograph of inkjet printing paper. (**f**) Inkjet printing paper under a 365 nm handheld UV lamp.

To get an understanding of the photophysical properties of IN-GQDs, UV-visible absorption and PL spectra analyses were carried out. Figures 6a and S6 show blue PL of the IN-GQDs and ON-GQDs, respectively, under illumination with a handheld 365 nm UV lamp. Figure 6b shows that an absorption peak of the IN-GQDs was detected at about 260 nm in the UV region due to the π → π* transition of benzene^8^,^49^. The other significant absorption peak at around 365 nm indicates the n → π* transition^3^,^50^,^51^. Apart from that, the PL properties of the IN-GQDs were observed using an adjustable UV-vis wavelength as an excitation source. When the excitation wavelength changed from 320 to 480 nm, the PL peaks of the IN-GQDs exhibited a red shift from 425 to 530 nm as shown in Fig. 6c. The highest intensity of the PL was observed for the IN-GQDs at 425 nm when excited at 360 nm. The PL properties of the IN-GQDs could be attributed to multiple factors including their size, shape, synthesis route, and surface modification^3^,^9^,^12^. Using quinine sulphate as a reference, the PL quantum yield (QY) of the IN-GQDs was calculated to be 34%, which is higher than that of the luminescent graphene-based quantum dots of previous studies^2^,^9^,^14^,^26^,^28^,^37^,^49^,^52^. Interestingly, it can be seen in Table 1 that the ON-GQDs exhibited a record-high QY of nearly 20% with the organic solvent of toluene. The IN-GQDs were very stable under irradiation with 365 nm UV light (Fig. 6d), indicating that the IN-GQDs have negligible photobleaching behavior, which means they may be one of the potential dye candidates for biological applications. In making use of the PL properties, the IN-GQDs were utilized as ink for inkjet printing patterns. A filtration paper (which exhibited no UV fluorescence response) to which the IN-GQDs adhered very well was selected as the printing paper. An aqueous solution of the IN-GQDs was injected into an empty cartridge taken from a commercial inkjet printer. Figure 6e shows no observable print on the paper after the IN-GQDs were used for printing. However, words and images were made visible with UV irradiation (Fig. 6f), which is an advantage for future practical applications.

### Application of IN-GQDs for non-volatile bio-memristor

**Figure 7:**
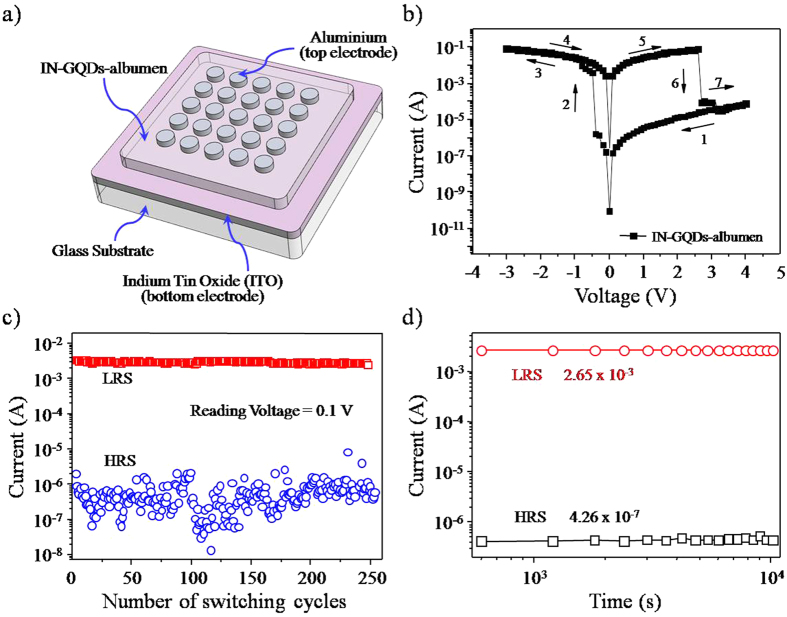
(**a**) Schematic illustration of non-volatile bio-memristor. (**b**) I-V characteristics of IN-GQDs-albumen device. The arrows show the sweeping direction of the bias voltage applied. (**c**) Endurance and (**d**) retention properties of the IN-GQDs-albumen device.

Recently, several protein-based materials were successfully utilized to fabricate a non-volatile bio-memristor. However, the performance of the bio-memristor still needed improvement in such aspects as switching and reliability. Thus, the hydrophilic behavior of the IN-GQDs inspired us to mix them with albumen for the first time to show the potential application of non-volatile bio-memristors. Figure 7a shows a schematic of IN-GQDs hybrid albumen memory devices on an indium tin oxide (ITO) electrode. The device was constructed as an ITO (bottom electrode)/IN-GQDs-albumen (insulator)/Al (top electrode) combination. The current-voltage (I-V) characteristics of the resistive memories fabricated with thin IN-GQDs-albumen films are shown in Fig. 7b. The fabricated devices exhibited a typical bi-stable state voltage ranging from −0.4 to 2.6 V to record the high resistance state (HRS) and low resistance state (LRS) signals of the memory, as shown in Figure S7 and summarized in Table S2. Based on previous studies on hybrid memory devices with nanoparticle- or quantum dot-incorporated active layers^53^,^54^, the enhancement of the bi-stable switching behavior of the device we used compared to that of the previously reported albumen-only device can be attributed to charge trapping and detrapping of the IN-GQDs as well as to the formation and rupture of filaments in the albumen matrix. In our device configuration, the electron was expected to be injected from an Al electrode under reverse bias and directly captured by the IN-GQDs, while the field being enhanced by the space charges facilitated the formation of filaments. Therefore, a very low set voltage (−0.4 V) was obtained. When the forward bias voltage exceeding 2.6 V was applied, the release of trapped electrons and the rupture of filaments caused a decrease in the number of conductive paths, and consequently, the device returned to its original HRS. Differences between V_SET_ and V_RESET_ of the fabricated devices were great enough to ensure that the HRS and LRS were perfectly distinguishable. The average V_SET_ value of the IN-GQDs-albumen device was −0.5 V, while the average V_RESET_ value was 2.5 V. This indicates that the ratio of signal to noise (S/N) and the capability of reading memory states exhibited superior performance to the previously-reported bio-molecule^55^. The maximum current on/off ratio between LRS and HRS for the IN-GQDs-albumen devices at an applied voltage of 1 V was greater than 10^4^, which is sufficient to reduce the probability of misreading^55^. Figure 7c shows the endurance test performed on the IN-GQDs-albumen memory device. The measurement results show that superior rewritable characteristics were obtained in the ITO/IN-GQDs-albumen/Al memory combination with a switching cycle of over 250 times. To evaluate retention efficiency, the current values of the HRS and LRS were recorded under a reading voltage of 0.1 V. Figure 7d shows the currents of the bio-memristor devices after writing and reading adjustments could be distinguished and maintained for more than 10^4 ^s. Table S3 summarizes the state-of-the-art operational parameters of the non-volatile bio-memristor. The retention and endurance properties of the IN-GQDs with an albumen bio-memristor are proven by the excellent performance, which makes them reliable and useful for non-volatile memory applications.

### Application of IN-GQDs for cellular imaging

**Figure 8:**
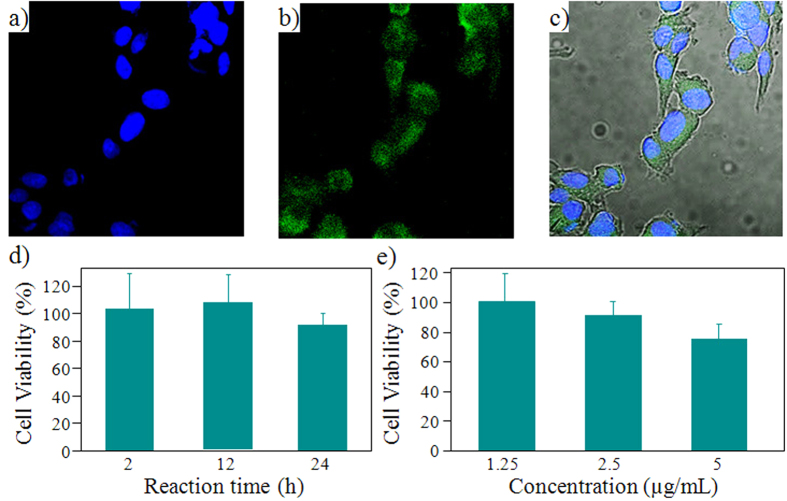
Two-color confocalPL cell imaging and cytotoxicity assessment of IN-GQDs using HeLa cells. The blue PL originated from nucleus counterstain Hoechst 34580, and the green PL was from IN-GQDs at an excitation wavelength at 408 nm. (**a**) Image of nucleus. (**b**) Image of microtubules. (**c**) Their corresponding PL overlaid with panels (**a,b**). (**d**) Reaction time vs. cellular cytotoxicity assessment. (**e**) IN-GQDs concentrations vs. cell viability.

Considering the facile procedure and intriguingly strong PL of the IN-GQDs, investigating whether IN-GQDs can be utilized as imaging probes for living cell imaging was worthwhile. To demonstrate the feasibility of the cellular imaging probe, the IN-GQDs were introduced into HeLa cells through endocytosis and then imaged by laser scanning confocal microscopy. After cell incubation with IN-GQDs for 4 h, the cells were washed thoroughly with phosphate buffered saline (PBS) to remove free IN-GQDs in the solution and nonspecific adsorption on the cell membrane. Figure 8b shows that IN-GQDs were distributed perinuclearly by using a laser excitation of 408 nm. The overlapped fluorescence and bright-field images (Fig. 8c) reveal that the signals of IN-GQDs fluorescence originated from the perinuclear regions of the cytosol, thus demonstrating a good degree of cell-permeability of the IN-GQDs into living HeLa cells. To get an understanding of the compatibility between cells and IN-GQDs, a cytotoxicity study was carried out with a conventional methylthiazolyldiphenyl-tetrazolium bromide (MTT) assay with HeLa cells. While dosing 2.5 μg/mL of IN-GQDs for 2, 12 and 24 h with HeLa cells, nearly 100% cell viability was observed, as shown in Fig. 8d. Therefore, we chose the reaction time of 24 h to further observe cell viability as a function of IN-GQDs concentrations. Relatively high cell viability was achieved after incubating HeLa cells with IN-GQDs at a concentration of 5 μg/mL, as shown in Fig. 8e. Thus, fluorescent IN-GQDs can be considered to be biocompatible and exhibit no toxicity for bio-imaging applications.

## Conclusion

In conclusion, we have demonstrated a facile and environmentally-friendly route for direct synthesis of ON-GQDs and IN-GQDs via exfoliating and disintegrating graphite flakes. To the best of our knowledge, this is the first study to show that ON-GQDs and IN-GQDs can be synchronized obtained via one-pot hydrothermal treatment. Various spectroscopic analyses including TEM, TM-AFM, FTIR, PL, XPS, and Raman demonstrated that the IN-GQDs and ON-GQDs originated from pyridinium-assisted exfoliated graphene sheets and undissolvable graphite flakes, respectively. The ON-GQDs/SWCNTs composite paper electrode exhibited an areal capacitance of 114 mF/cm^2^, which is nearly three orders of magnitude higher than reduced graphene oxide devices, and it had excellent cycling stability. The hybrid IN-GQDs-albumen memory devices exhibited on/off current ratios of higher than 10^4^ accompanied by a reliable switching endurance of over 250 cycles. Moreover, the HRS and LRS could be maintained over a long period of time (>10^4 ^s). The IN-GQDs had several advantages, such as a superior QY of 34%, excellent stability of cellular imaging, and no cytotoxicity. The ease of preparation could make it a promising method for using IN-GQDs and ON-GQDs in numerous applications, such as carbon-based supercapacitors, non-volatile bio-memories, and bio-imaging.

## Methods

### Materials

Graphite powder was supplied by Sigma-Aldrich. Pyridinium tribromide (PyBr_3_, TCI) and polyethylenimine branched (PEI, Mw ~25,000, Sigma-Aldrich) were used as received. Single-walled carbon nanotubes (SWCNTs) powder was obtained from SouthWest Nanotechnologies Inc. Triton X-100 were purchased from Golden Innovation Business Co. Ltd. and used for SWCNTs’ dispersion. Analytical grade toluene, Dimethyl sulfoxide (DMSO) and ethanol were purchased from Sigma-Aldrich and used without further purification. The HeLa cell lines were purchased from Food Industry Research and Development Institute (Taiwan). The HeLa cells were grown in the environment of Dulbecco’s Modified Eagle Medium (cat. no. 11885, Invitrogen) at 37 °C with 5% CO_2_ humidified atmosphere.

### Hydrothermal synthesis

Graphite powder (5 mg) was added to a pyridinium tribromine-contained aqueous solution: ethanol (1:1) solution (20 mL, 1 M). Then, the well-stirred solution was sonicated for 1 h. The corresponding solution containing exfoliated monolayer graphene sheets and undissolvable flakes was prepared, and then, PEI was added and stirred until it dissolved. The PyBr_3_ and the PEI played the roles of graphene exfoliant and doping source of nitrogen atoms, respectively. The fully mixed solution was transferred to a stainless autoclave reactor at 230 °C for 8 h. The resulting solution of the IN-GQDs and ON-GQDs was formed via chemical functionalization^26^,^43^, filtered by syringe membrane filters (0.45 μm), and then centrifuged at 12,000 rpm for 30 min to collect a high-concentration IN-GQDs and ON-GQDs solution. The IN-GQDs and ON-GQDs were diluted with water and toluene, respectively, and kept at room temperature for further use.

### Material characterizations

The optical data of the IN-GQDs and ON-GQDs were measured in liquid cells made of quartz. The PL spectra were measured using a PL spectrophotometer (Hitachi F-4500, Japan) at room temperature. The absorbance was recorded at ambient conditions on a Unicam UV-300 UV–vis spectrophotometer. FTIR spectroscopy was recorded using a Perkin Elmer Frontier spectrometer with an attenuated total reflection (ATR) attachment in the range of 700–4000 cm^−1^. A TM-AFM (Innova/Bruker, Santa Barbara, CA) was utilized to measure the thickness for the IN-GQDs and ON-GQDs. The TM-AFM tips (NSC15/AI BS) with nominal spring constant of 40 N/m were purchased from MikroMasch, CA, USA. The XPS measurements were carried out by using a Thermo K-Alpha (VGS) with Al Kα X-ray (1486.6 eV) as a radiation source. The measured XPS spectra were calibrated with Au 4f_7/2_ at 84.0 eV. The IN-GQDs and ON-GQDs were characterized using TEM (JEOL JEM-2100, Japan). Raman spectra were recorded using a iHR550 spectrometer (Horiba Jobin Yvon Inc., NJ) with an excitation laser source of 532 nm under ambient conditions; the power of the laser was set below 100 mW, the spot-size of the laser was approximately 5 μm, and the peak of Si at 520.7 cm^−1^ was used for calibration. Electrochemical studies were measured using a CHI 7279E (CH instrument Corp.). The electrical contacts for the ON-GQDs/SWCNTs paper electrode were connected using alligator clip.

### Preparation of ON-GQDs/SWCNTs hybrid composite papers and their electrochemical measurements

Firstly, the working electrodes were fabricated by dispersing SWCNTs powder in deionized water (40 mg/L) with a few drops of Triton X-100 surfactant, then sonicated for 1 h. After using filtration equipment to filter the SWCNTs suspension, the working electrodes for the SWCNTs papers were made. The SWCNTs papers were heated to 200 °C to remove the surfactant of Triton X-100. Then, the SWCNTs papers were immersed in the ON-GQDs solution for 24 h, and ON-GQDs were subsequently adsorbed onto the surface of the SWCNTs via π-π interactions. The ON-GQDs/SWCNTs paper electrode was cut into rectangles of 1 cm × 0.5 cm in size and directly utilized as working electrodes in a three-electrode system with Ag/AgCl as the reference electrode and Pt wire as the counter electrode. All electrochemical tests were carried out using a CHI 7279E electrochemistry workstation with a potential of −0.4 to 0.6 V in 1 M KOH electrolyte at room temperature.

### Bio-memristor fabrication process and device configuration

Chicken eggs were purchased from traditional market. The albumen liquid was carefully isolated from the chicken egg by drilling a hole via the shell of an egg. Then, the IN-GQDs and the albumen liquid were taken in the weight ratio of 1:5 and fully blended for 5 min. An indium tin oxide (ITO)-coated substrate were thoroughly washed detergent, water, acetone and 2-propanol in an ultrasonication water bath for 20 min. The ITO layer acted as the bottom electrode. The liquid of the IN-GQDs-albumen was spin-coated on the ITO-coated glass substrate with a spin speed of 5000 rpm for 40 s. The IN-GQDs-albumen-coated ITO substrates were baked at high temperature in ambient conditions and dried at room temperature in a vacuum chamber, respectively. The baking condition was started gradually from 100 °C to 120 °C and then to 140 °C, being maintained for 10 min at each stage. To construct the device structure, a thickness of 150 nm aluminum (Al) films were thermally evaporated on the device through a 1 × 1 mm^2^ square patterns mask as shown in Fig. 7a. The Al and ITO films served as top and bottom electrodes, respectively.

### Cell imaging

After cell incubation with IN-GQDs for 4 h, the cells were washed thoroughly with PBS to remove free IN-GQDs in solution and nonspecific adsorption on the cell membrane. The fluorescence spectra of IN-GQDs tagged cells were received with a fluorescence confocal microscope (Nikon D-Eclipse C1) under atmospheric conditions. A diode laser at 408 nm (~15 mW) as the excitation source and an integration time of 1.6 μs/pixel was used to collect the confocal fluorescence images. An oil objective (CF1 Plan Fluor 100×) was used for cell-imaging and spectral data acquisition; the laser spot size was ~7 μm^2^.

### Cytotoxicity assay

The cell viability of the IN-GQDs was examined using HeLa cells. The total amount of viable cells was expressed using the MTT assay with 3-(4,5-dimethylthiazole-2-yl)-2,5-phenyltetrazolium bromide. HeLa cells were first cultured in a 24 well culture plate and then incubated with various concentrations of IN-GQDs (1.25, 2.5, 5.0 μg/mL) for 6, 12, and 24 h. After incubating, MTT aqueous solution (20 μL, 5 mg/mL) was added to each well, and the HeLa cells were incubated another 4 h at 37 °C to deoxidize the MTT. The medium was then thoroughly washed out, and DMSO was added 300 μL to each well to dissolve the formazan crystals. ABioTek ELx800 microplate reader was used to measure the absorbance at 570 nm, while the control experiment contained cells cultured with pure medium (e.g., without IN-GQDs).

## Additional Information

**How to cite this article**: Kuo, N.-J. *et al*. One-pot synthesis of hydrophilic and hydrophobic N-doped graphene quantum dots via exfoliating and disintegrating graphite flakes. *Sci. Rep.* **6**, 30426; doi: 10.1038/srep30426 (2016).

## Supplementary Material

Supplementary Information
