# Supplementary material for: One-Pot Synthesis of Hydrophilic and Hydrophobic N-Doped Graphene Quantum Dots via Exfoliating and Disintegrating Graphite Flakes
Source: Sci Rep. 2016 Jul 25;6:30426. doi: 10.1038/srep30426 (PMC4958986; doi:10.1038/srep30426)
Supplement: Supplementary Information [file srep30426-s1.doc]

Supplementary Information

One-Pot Synthesis of Hydrophilicity and Hydrophobicity N-Doped Graphene Quantum Dots via Exfoliating and Disintegrating Graphite Flakes

Na-Jung Kuo^1^*,* Yu-Syuan Chen^1^, Chien-Wei Wu^2^*,* Chun-Yuan Huang^1^, Yang-Hsiang Chan^3^ & I-Wen Peter Chen^1,*^

^1^ Department of Applied Science, National Taitung University, 369, Sec. 2, University Road., Taitung City 95092 (Taiwan)

^2^ Department of Chemistry, National Taiwan University, 1, Sec. 4, Roosevelt Road, Taipei, 10617 (Taiwan)

^3^ Department of Chemistry, National Sun Yat-sen University, 70 Lien Hai Road, Kaohsiung, 80424 (Taiwan)

^*^Corresponding. iwchen@nttu.edu.tw

Contents

The solution of the pyridinium-assisted exfoliated graphene sheets........................ 1

Variation of centrifugation speeds............................................................................. 2

Statistical thickness distributions of the IN-GQDs and ON-GQDs......................... 3

FTIR spectrum of pure PEI....................................................................................... 4

The capacitance of the pure SWCNT paper electrode.............................................. 5

PL image of the ON-GQDs....................................................................................... 6

V_SET_ and V_RESET_ distributions of IN-GQDs-albumen device................................. 7

Quantum yield of the IN-GQDs and ON-GQDs....................................................... 8

Performance comparison of albumen and IN-GQDs-albumen memory devices..... 9

Performance comparison of bio-memristor devices................................................. 10

**The solution of the pyridinium-assisted exfoliated graphene sheets**


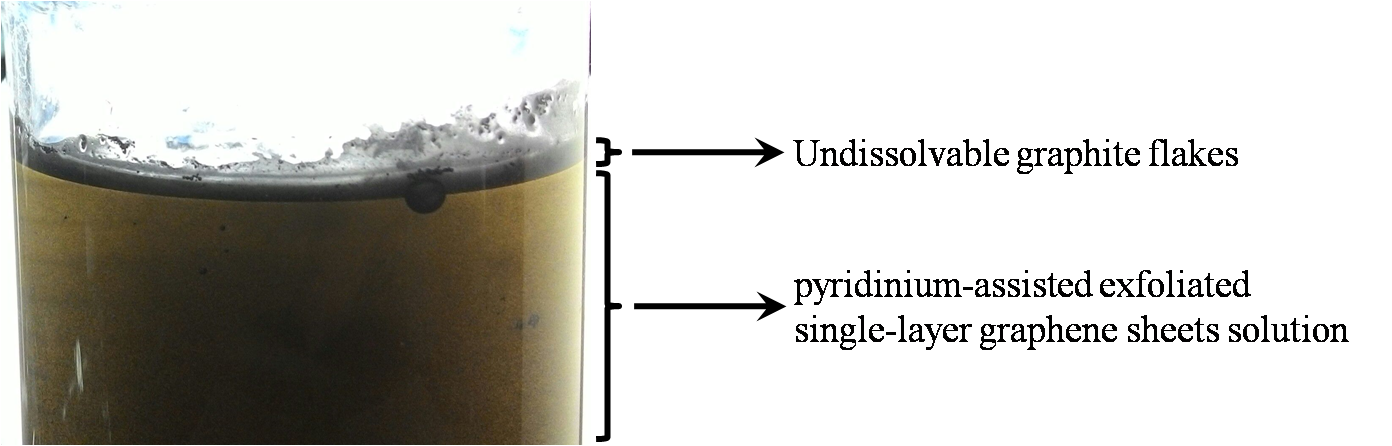


Figure S1 Suspension of pyridinium-assisted exfoliated graphene sheets

**Variation of centrifugation speeds**

Figure S2. The as-prepared IN-GQDs under various centrifugation speeds for 30 min.

**Statistical thickness distributions of the IN-GQDs and ON-GQDs**

Figure S3. Statistical thickness of the N-GQDs distributions measured by TM-AFM on a) 591 sample dots of the IN-GQDs and b) 104 sample dots of the ON-GQDs.

**FTIR spectrum of pure PEI.**


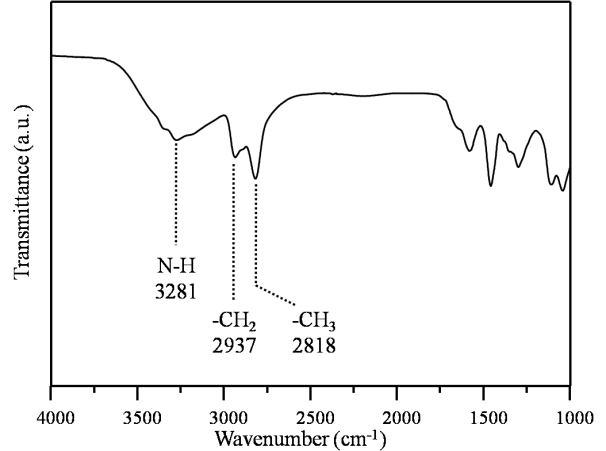


Figure S4. FTIR spectrum of pure PEI.

**The capacitance of the pure SWCNT paper electrode**

Figure S5. The capacitance of the pure SWCNT paper electrode.

**PL image of the ON-GQDs**


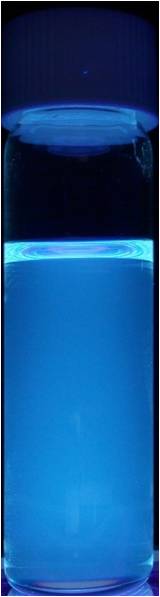


Figure S6. PL of the ON-GQDs under illumination with a handheld 365 nm UV lamp.

**V_SET_ and V_RESET_ distributions of IN-GQDs-albumen device**


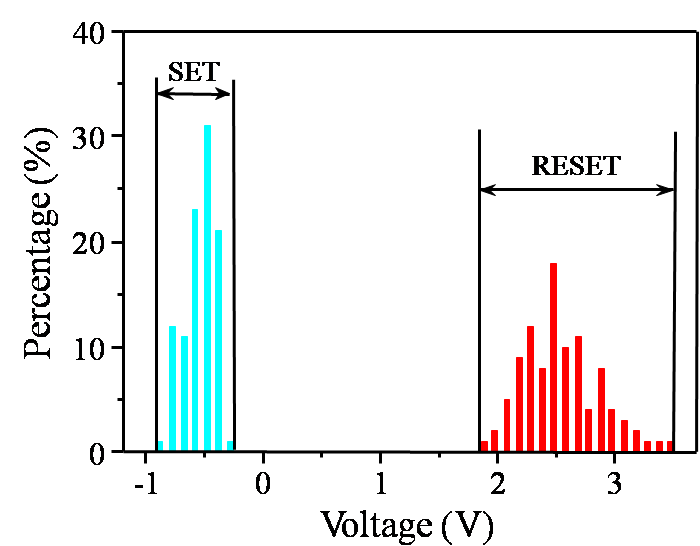


Figure S7. V_SET_ and V_RESET_ distributions of IN-GQDs-albumen device.

**Quantum yield of the IN-GQDs and ON-GQDs**

Table S1. Quantum yield of the IN-GQDs and ON-GQDs.

| Sample | Integrated Emission Intensity (*I*) | Absorbance at 350 nm (*A*) | Refractive Index of Solvent (*η*) | Quantum Yield (*Ф*) |
| --- | --- | --- | --- | --- |
| Quinine sulphate | 663928.864 | 0.0852 | 1.33 | 54% |
| IN-GQDs | 233182.2655 | 0.0487 | 1.3477 | 34.07% |
| ON-GQDs | 142476.08 | 0.0625 | 1.495 | 19.96% |

**Performance comparison of albumen and IN-GQDs-albumen memory devices**

Table S2. Performance comparison of albumen and IN-GQDs-albumen devices.

| Sample | Read (V) | Write (V) | Erase (V) | On (A) | Off (A) | On/Off |
| --- | --- | --- | --- | --- | --- | --- |
| albumen | 0.1 | -0.6 | 2.8 | 2.49×10^-3^ | 1.19×10^-6^ | 2.08×10^3^ |
| IN-GQDs-albumen | 0.1 | -0.4 | 2.6 | 2.5×10^-3^ | 1.55×10^-7^ | 1.61×10^4^ |

**Performance comparison of bio-memristor devices**

Table S3. Performance comparison of bio-memristor devices.

| Devices | Memory Property | | | References |
| --- | --- | --- | --- | --- |
|  | I_on/off_ | Retention time (s) | Switching cycles |  |
| Silk fibroin protein | ~ 10 | > 800 | ~ 120 | [^1^](#_ENREF_1) |
| DNA | ~30 | > 10^5^ | 100 | [^2^](#_ENREF_2) |
| Enzyme multilayers | > 10^2^ | > 10^4^ | ~ 200 | [^3^](#_ENREF_3) |
| Silk fibroin protein/Au nanoparticles blends | > 10^6^ | > 10^2^ | > 10 | [^4^](#_ENREF_4) |
| Sericin | ~10^6^ | > 10^3^ | ~ 21 | [^5^](#_ENREF_5) |
| Al-chelated gelatine | >10^4^ | > 10^4^ | ~ 60 | [^6^](#_ENREF_6) |
| Myoglobin | >10^4^ | > 10^2^ | ~ 100 | [^7^](#_ENREF_7) |
| Tobacco mosaic virus | ~ 500 | > 10^3^ | 200 | [^8^](#_ENREF_8) |
| IN-GQD-albumen | >10^4^ | > 10^4^ | > 250 | This work |

References:

1. Hota, M. K.*et al.* A Natural Silk Fibroin Protein-Based Transparent Bio-Memristor. *Adv. Funct. Mater.* **22**, 4493-4499 (2012).

2. Qin, S.; Dong, R.; Yan, X.; Du, Q. A Reproducible Write-(Read)n-Erase and Multilevel Bio-Memristor Based on DNA Molecule. *Org. Electron.* **22**, 147-153 (2015).

3. Baek, H.; Lee, C.; Lim, K.-i.; Cho, J. Resistive Switching Memory Properties of Layer-by-Layer Assembled Enzyme Multilayers. *Nanotechnology* **23**, 155604 (2012).

4. Gogurla, N.*et al.* Transparent and Flexible Resistive Switching Memory Devices with a Very High ON/OFF Ratio using Gold Nanoparticles Embedded in a Silk Protein Matrix. *Nanotechnology* **24**, 345202 (2013).

5. Wang, H.*et al.* Sericin for Resistance Switching Device with Multilevel Nonvolatile Memory. *Adv. Mater.* **25**, 5498-5503 (2013).

6. Chang, Y.-C.; Wang, Y.-H. Solution-Processed Al-Chelated Gelatin for Highly Transparent Non-Volatile Memory Applications. *Appl. Phys. Lett.* **106**, 123302 (2015).

7. Lee, J. H.; Yew, S. C.; Cho, J.; Kim, Y. S. Effect of Redox Proteins on the Behavior of Non-Volatile Memory. *Chem. Commun.* **48**, 12008-12010 (2012).

8. Tseng, R. J.*et al.* Digital Memory Device Based on Tobacco Mosaic Virus Conjugated with Nanoparticles. *Nat. Nanotechnol.* **1**, 72 (2006).
